# Growth-Inhibiting and morphostructural effects of constituents identified in *Asarum heterotropoides* root on human intestinal bacteria

**DOI:** 10.1186/1472-6882-13-245

**Published:** 2013-10-01

**Authors:** Haribalan Perumalsamy, Moon Young Jung, Seung Min Hong, Young-Joon Ahn

**Affiliations:** 1 Research Institute for Agricultural and Life Sciences, College of Agriculture and Life Sciences, Seoul National University, Seoul 151-921, Republic of Korea; 2 Department of Agricultural Biotechnology, WCU Biomodulation Major, Seoul National University, Seoul 151-921, Republic of Korea

**Keywords:** *Asarum heterotropoides*, Natural growth inhibitor, Gram-positive bacteria, Gram-negative bacteria, Morphological alteration, Minimal inhibitory concentrations

## Abstract

**Background:**

The growth-inhibiting and morphostructural effects of seven constituents identified in *Asarum heterotropoides* root on 14 intestinal bacteria were compared with those of the fluoroquinolone antibiotic ciprofloxacin.

**Method:**

A microtiter plate-based bioassay in sterile 96-well plates was used to evaluate the minimal inhibitory concentrations (MICs) of the test materials against the organisms.

**Results:**

δ-3-Carene (**5**) exhibited the most potent growth inhibition of Gram-positive bacteria (*Clostridium difficile* ATCC 9689, *Clostridium paraputrificum* ATCC 25780, *Clostridium perfringens* ATCC 13124, and *Staphylococcus aureus* ATCC 12600) and Gram-negative bacteria (*Escherichia coli* ATCC 11775 and *Bacteroides fragilis* ATCC 25285) (minimal inhibitory concentrations (MIC), 0.18–0.70 mg/mL) except for *Salmonella enterica* serovar Typhimurium ATCC 13311 (MIC, 2.94 mg/mL). The MIC of methyleugenol (**2**), 1,8-cineole (**3**), α-asarone (**4**), (−)-asarinin (**6**), and pellitorine (**7**) was between 1.47 and 2.94 mg/mL against all test bacteria (except for compound **2** against *C. difficile* (0.70 mg/mL); compounds **1** (23.50 mg/mL) and **4** (5.80 mg/mL) against *C. paraputricum*; compounds **2** (5.80 mg/mL), **4** (12.0 mg/mL), and **7** (0.70 mg/mL) against *C. perfringens*); compound **1** against *E. coli* (7.20 mg/mL) and *S. enterica* serovar Typhimurium (12.0 mg/mL). Overall, all of the constituents were less potent at inhibiting microbial growth than ciprofloxacin (MIC, 0.063–0.25 mg/ mL). The lactic acid-producing bacteria (four bifidobacteria and two lactobacilli) and one acidulating bacterium *Clostridium butyricum* ATCC 25779 were less sensitive and more susceptible than the five harmful bacteria and two nonpathogenic bacteria (*B. fragilis* and *E. coli*) to the constituents and to ciprofloxacin, respectively. Beneficial Gram-positive bacteria and harmful and nonpathogenic Gram-negative bacteria were observed to have different degrees of antimicrobial susceptibility to the constituents, although the antimicrobial susceptibility of the harmful Gram-positive bacteria and the harmful and nonpathogenic Gram-negative bacteria was not observed. Scanning electron microscopy observations showed different degrees of physical damage and morphological alteration to both Gram-positive and Gram-negative bacteria treated with α-asarone, δ-3-carene, pellitorine, or ciprofloxacin, indicating that they do not share a common mode of action.

**Conclusion:**

*A. heterotropoides* root-derived materials described merit further study as potential antibacterial products or lead molecules for the prevention or eradication from humans from diseases caused by harmful intestinal bacteria.

## Background

In humans, various microorganisms reside in the gastrointestinal tract as a highly complex ecosystem with considerable species diversity (approximately 500–1,000 bacterial species) [1]. Gastrointestinal ecological investigations have indicated that the normal microbiota is predominantly composed of lactic acid-producing bacteria, whereas the microbiota of cancer patients or elderly subjects is mainly composed of *Clostridium* with only few lactic acid-producing bacteria [2-4]. The microbiota participate in normal physiological functions, including metabolic activities that result in salvage of energy and absorbable nutrients, and important trophic effects on intestinal epithelia and on immune structure and function [5,6]. It also contribute to the genesis of various disease states by biotransforming a variety of ingested or endogenously formed compounds to potentially harmful metabolites such as *N*-nitroso compounds or aromatic steroids [6-8]. Prolonged treatment with antibiotics alters the normal microbial population of the gastrointestinal tract, eliminating some of the beneficial bacteria [4] and often producing resistance to the drugs by pathogenic microorganisms [8,9], which is a major global public health problem in both developed and developing countries. Sometimes, serious side effects of antibiotics occur, such as taste disturbances, nausea, diarrhea, dyspepsia, headache, and angioedema [10]. There is, therefore, a critical need for the development of new improved antibacterial agents with novel target sites and low toxicity.

Plant secondary substances may provide potential sources of growth modulators against gastrointestinal bacteria largely because higher plants constitute a potential source of bioactive chemicals that have been perceived by the general public as relatively safe and with minimal impacts to human health [11-13]. They can act at multiple and novel target sites, thereby reducing the potential for resistance [14]. Much effort has been focused on plant preparations as potential sources of commercial growth promoters of beneficial intestinal bacteria and/or growth inhibitors of harmful intestinal bacteria. In the screening of plants for antibacterial activity, a methanol extract of *Asarum heterotropoides* (Aristolochiaceae) was shown to have growth inhibitory activity against *Bacteroides fragilis* ATCC 25285 and *Clostridium perfringens* ATCC 13124. Historically, *A. heterotropoides* has long been used as an analgesic and antitussive agent for the treatment of influenza, headache, rheumatic pain, and asthma [15]. No information has been obtained concerning the potential of *A*. *heterotropoides* root-derived materials to modulate human gastrointestinal bacteria, despite its excellent pharmacological action, such as antiallergic, antihyperlipdemic, liver protective, antihistamine, and antimicrobial activities [15].

In the current study, an assessment is made of the growth inhibitory activity of seven constituents identified in *A*. *heterotropoides* root against five harmful intestinal bacteria, two nonpathogenic intestinal bacteria, and seven beneficial intestinal bacteria (six lactic acid-producing bacteria and one acidulating bacterium), including Gram-positive bacteria and Gram-negative bacteria, using a microtiter plate-based bioassay. The growth inhibitory activity of the constituents was compared with that of ciprofloxacin, a second-generation fluoroquinolone antibiotic with a broad spectrum [16]. The effects of the constituents on morphological changes of the bacterial strains were examined using scanning electron microscopy (SEM). The possible mode of action of the constituents is also discussed.

## Methods

### Instrumental analyses

^1^H and ^13^C NMR spectra were recorded in CDCl_3_ or MeOD on a Bruker AVANCE 600 spectrometer (Karlsruhe, Germany) using tetramethylsilane as an internal standard, and chemical shifts are given in δ (ppm). UV spectra were obtained in ethanol on a Kontron UVICON 933/934 spectrophotometer (Milan, Italy), FT-IR spectra on a Thermo Scientific Nicolet 6700 FT-IR (Madison, WI), and mass spectra on a Jeol JMS-DX 303 spectrometer (Tokyo, Japan). Optical rotation was measured with a Rudolph Research Analytical Autopol III polarimeter (Flanders, NJ, USA). Merck silica gel (0.063–0.2 mm) (Darmstadt, Germany) was used for column chromatography. Merck precoated silica gel plates (Kieselgel 60 F_254_) were used for analytical thin-layer chromatography (TLC). Merck preparative silica gel plates and an Agilent 1200 series high-performance liquid chromatography (HPLC) (Santa Clara, CA, USA) were used for isolation of active principles.

### Plant sample

Air-dried root (600 g) of *A. heterotropoides* was purchased from Boeun medicinal herb shop, Kyoungdong market (Seoul, South Korea). It was identified by Dr. Sang-Cheol Shin, Korea Forest Research Institute. A voucher specimen (AHR-01) was deposited in the Research Institute for Agriculture and Life Science, Seoul National University.

### Bacterial strains and culture conditions

Five harmful intestinal bacteria, two nonpathogenic intestinal bacteria, six lactic acid-producing intestinal bacteria, and one acidulating bacterium examined in this study are listed in Table 1. They were purchased from the American Type Culture Collection (ATCC). Stock cultures of these bacterial strains were routinely stored on BHI broth (Becton, Dickinson and Company, Sparks, MD, USA) as reported previously [17], when required were subcultured on BHI broth (pH 7.6). The cultures were incubated at 37°C for 1 day in an atmosphere of 5% H_2_, 15% CO_2_, and 80% N_2_ in a Hirayama anaerobic chamber (Tokyo, Japan), except for the cultures of *Escherichia coli* and *Staphylococcus aureus* which were incubated at 37°C for 1 day under aerobic condition. For bioassay, bacterial suspensions containing 1 × 10^5^ colony-forming unit (CFU)/mL were prepared in EG agar (Eiken Chemical, Tokyo) using the 24 h subcultures in BHI broth. The cell density was estimated by measuring the turbidity.

**Table 1:** List of human intestinal bacteria tested for growth inhibitory activity

**Harmful or nonpathogenic intestinal bacteria**	**Beneficial intestinal bacteria**
Gram-positive	Gram-positive
*Clostridium difficile* ATCC 9689^a^	*Bifidobacterium bifidum* ATCC 29521^c^
*Clostridium paraputrificum* ATCC 25780^a^	*Bifidobacterium breve* ATCC 15700^c^
*Clostridium perfringens* ATCC 13124^a^	*Bifidobacterium infantis* ATCC 25962^c^
*Staphylococcus aureus* ATCC 12600^a^	*Bifidobacterium longum* ATCC 15707^c^
	*Lactobacillus acidophilus* ATCC 4356^c^
	*Lactobacillus casei* ATCC 393^c^
	*Clostridium butyricum* ATCC 25779^d^
Gram-negative	
*Bacteroides fragilis* ATCC 25285^b^	
*Escherichia coli* ATCC 11775^b^	
*Salmonella enterica* serovar Typhimurium ATCC 13311^a^	

^a^Harmful bacteria.

^b^Nonpathogenic bacteria.

^c^Lactic acid-producing bacteria.

^d^Acidulating bacterium.

### Extraction and isolation

Extraction procedures of air-dried root of *A. heterotropoides* were performed as reported previously [18]. The hexane-soluble fraction (10 g) was most active and was chromatographed on a 70 × 5.5 cm silica gel column (600 g) and eluted with a gradient of hexane and ethyl acetate [(10:1 (2.2 L), 9:1 (2 L), 7:3 (2 L), 5:5 (1 L), and 3:7 (1 L) by volume] and finally with methanol (1 L) to provide 48 fractions (each about 250 mL). Column fractions were monitored by TLC on silica gel plates with hexane and ethyl acetate (7:3 by volume). Fractions with similar *R*_*f*_ values on the TLC plates were pooled. Spots were detected by spraying with 2% H_2_SO_4_ and then heating on a hot plate. Fractions 1–4 (2.92 g) were purified by preparative TLC with hexane and ethyl acetate (7:3 by volume) to yield two active principles **1** (465 mg, *R*_*f*_ = 0.73) and **2** (720 mg, *R*_*f*_ = 0.94). Fractions 5–8 (450 mg) were purified by preparative TLC with hexane and ethyl acetate (7:3 by volume) to provide three active principles **3** (15 mg, *R*_*f*_ = 0.58), **4** (75 mg, *R*_*f*_ = 0.61), and **5** (23 mg, *R*_*f*_ = 0.63). Fractions 15–27 (760 mg) were recrystallized in methanol at −4°C to afford an active principle **6** (2.11 mg). A preparative HPLC was used for separation of the constituents from the active fractions 28–32 (739 mg). The column was a 21.2 mm i.d. × 250 mm Phenomenex Prodigy ODS (Torrance, CA) using a mobile phase of acetonitrile and water (8:2 by volume) at a flow rate of 1.5 mL/min. Chromatographic separations were monitored using a UV detector at 254 nm. Finally, an active principle **7** (12 mg) was isolated at a retention time of 10.89 min.

### Growth-inhibiting assay

A microtiter plate-based bioassay in sterile 96-well plates was used to evaluate the minimal inhibitory concentrations (MICs) of the test materials against the organisms [19]. In brief, initial test materials were prepared in dimethylsulfoxide (DMSO), and 2-fold serial dilutions were then performed in 50 μL EG broth. Subsequently, 10 μL bacterial suspension of each strain was added. Ciprofloxacin (Sigma-Aldrich, St. Louis, MO, USA) served as a positive control and were similarly prepared. Negative controls consisted of the DMSO solution. The plates were incubated at 37°C under anaerobic conditions in a Hirayama chamber as stated previously, except for the plates of *S. aureus* and *E. coli* which were incubated at 37°C under aerobic conditions. After 24 h incubation, 10 μL of resazurin solution (270 mg resazurin in 40 mL sterile distilled water) was added to each well.

### Scanning electron microscopy

Both chemical-untreated (control) and -treated bacterial samples were centrifuged at 3000 × *g* at 4°C for 5 min. The bacterial pellets were primarily fixed in Karnovsky’s fixative (2% glutaraldehyde (v/v) and 2% paraformaldehyde (v/v) in 0.05 M sodium cacodylate buffer pH 7.2) [20]. The samples were incubated at 4°C in darkness for 2–4 h. They were then washed three times with the same buffer. Second fixing was performed with 1% OsO_4_ (w/v) in the same buffer at 4°C for 2 h. Fixed samples were washed two times with the same buffer and distilled water. The samples were then dehydrated in a graded series of ethanol increasing concentrations up to 100% for 10 min. Finally, samples were substituted to hexamethyldisilazane and dried in a Bio-Rad E3000 critical point drying machine (Cambridge, MA, USA). Specimen was photographed using a Jeol JSM 5410LV scanning electron microscope (Tokyo) at 20 kV.

### Data analysis

MICs were defined as the lowest concentrations that visually inhibited bacterial growth using resazurin indicator. All bioassays were repeated three times in triplicate and standard error was 0–6% of the values. A constituents having MIC >120 mg/mL was considered to be ineffective. Bonferroni multiple-comparison method was used to test for significant differences among the treatments (SAS OnlineDoc, Version 8.01; SAS Institute, Cary, NC, USA).

## Results

### Bioassay-guided fractionation and identification

Fractions obtained from the solvent hydrolyzable of the methanol extract of *A. heterotropoides* root were tested against five harmful intestinal bacteria and two nonpathogenic intestinal bacteria by a microtiter plate-based bioassay (Table 2). Based on MIC values, the methanol extract was proved to have growth inhibitory activity against all of the intestinal bacteria. The hexane-soluble fraction showed the most potent growth inhibition, followed by the ethyl acetate- and chloroform-soluble fractions. Weak and no growth inhibition were produced by the butanol- and water-soluble fractions, respectively. The beneficial intestinal bacteria were less susceptible than the harmful intestinal bacteria to the root-derived materials, although the hexane-soluble fraction showed the most potent growth inhibition (Table 3). Therefore, the hexane-soluble fraction was used to identify peak activity fractions for the next step in the purification.

**Table 2:** **Minimal inhibitory concentrations (MICs) of fractions obtained from the solvent hydrolyzable of the methanol extract of *Asarum heterotropoides *root against harmful and nonpathogenic intestinal bacteria using a microtiter plate-based antibacterial bioassay**

		**MIC (mg/mL)**					
	**Gram-positive bacteria**^**b**^				**Gram-negative bacteria**^**c**^		
**Material**^**a**^	**Cd**	**Cm**	**Cp**	**Sa**	**Bf**^**d**^	**Ec**^**d**^	**St**
ME	14.70 bA	7.35 cB	7.35 cB	14.70 bA	7.35 bB	14.70 bA	7.35 bB
HSF	7.35 cB	14.70 bA	7.35 cB	7.35 cB	3.67 cC	7.35 cB	3.67 cC
CSF	14.70 bA	14.70 bA	14.70 bA	7.35 cB	7.35 bB	14.70 bA	7.35 bB
EASF	7.35 cB	7.35 cB	14.70 bA	7.35 cB	1.83 dC	14.70 bA	7.35 bB
BSF	29.40 aB	58.80 aA	29.40 aB	29.40 aB	58.80 aA	58.80 aA	14.70 aC
WSF	NA^e^	NA	NA	NA	NA	NA	NA

Means in a column followed by the same lowercase letter and means in a row followed by the same uppercase letter are not significantly different (P > 0.05; Bonferroni test).

^a^ME, methanol extract; HSF, hexane-soluble fraction; CSF, chloroform-soluble faction; EASF, Ethyl acetate-soluble fraction; BSF, butanol-soluble fraction; WSF, water-soluble fraction.

^b^Cd, *C. difficile* ATCC 9689; Cm, *C. paraputiricum* ATCC 25780; Cp, *C. perfringens* ATCC 13124; Sa, *S. aureus* ATCC 12600.

^c^Bf, *B. fragilis* ATCC 25285; Ec, *E. coli* ATCC 11775; St, *S. enterica* serovar Typhimurium ATCC 13311.

^d^Nonpathogenic bacteria.

^e^No activity.

**Table 3:** **Minimal inhibitory concentrations (MICs) of fractions obtained from the solvent hydrolyzable of the methanol extract of *Asarum heterotropoides *root against beneficial Gram-positive intestinal bacteria using a microtiter plate-based antibacterial bioassay**

	**MIC (mg/mL)**						
**Material**^**a**^	**Bb**^**b**^	**Bi**^**b**^	**Be**^**b**^	**Bl**^**b**^	**La**^**b**^	**Lc**^**b**^	**Cb**^**c**^
ME	60.67 aA	58.50 bB	58.50 aB	58.50 aB	29.30 bC	58.50 aB	58.50 aB
HSF	29.30 dB	29.30 cB	29.30 bB	29.30 bB	29.30 bB	58.50 aA	29.30 bB
CSF	55.87 cB	58.50 bA	58.50 aA	29.30 bC	29.30 bC	58.50 aA	58.50 aA
EASF	58.50 bB	117.0 aA	58.50 aB	58.50 aB	58.50 aB	55.87 bC	58.50 aB
BSF	NA^d^	NA	NA	NA	NA	NA	NA
WSF	NA	NA	NA	NA	NA	NA	NA

Means in a column followed by the same lowercase letter and means in a row followed by the same uppercase letter are not significantly different (P > 0.05; Bonferroni test).

^a^ME, methanol extract; HSF, hexane-soluble fraction; CSF, chloroform-soluble faction; EASF, Ethyl acetate-soluble fraction; BSF, butanol-soluble fraction; WSF, water-soluble fraction.

^b^Lactic acid-producing bacteria: Bb, *B. bifidum* ATCC 29521; Bi, *B. infantis* ATCC 25962; Be, *B. breve* ATCC 15700; Bl, *B. longum* ATCC 15707; La, *L. acidophilius* ATCC 4356; Lc, *L. casei* ATCC 393.

^c^Acidulating bacteria: Cb, *C. butyricum* ATCC 25779.

^d^No activity.

**Figure 1:**
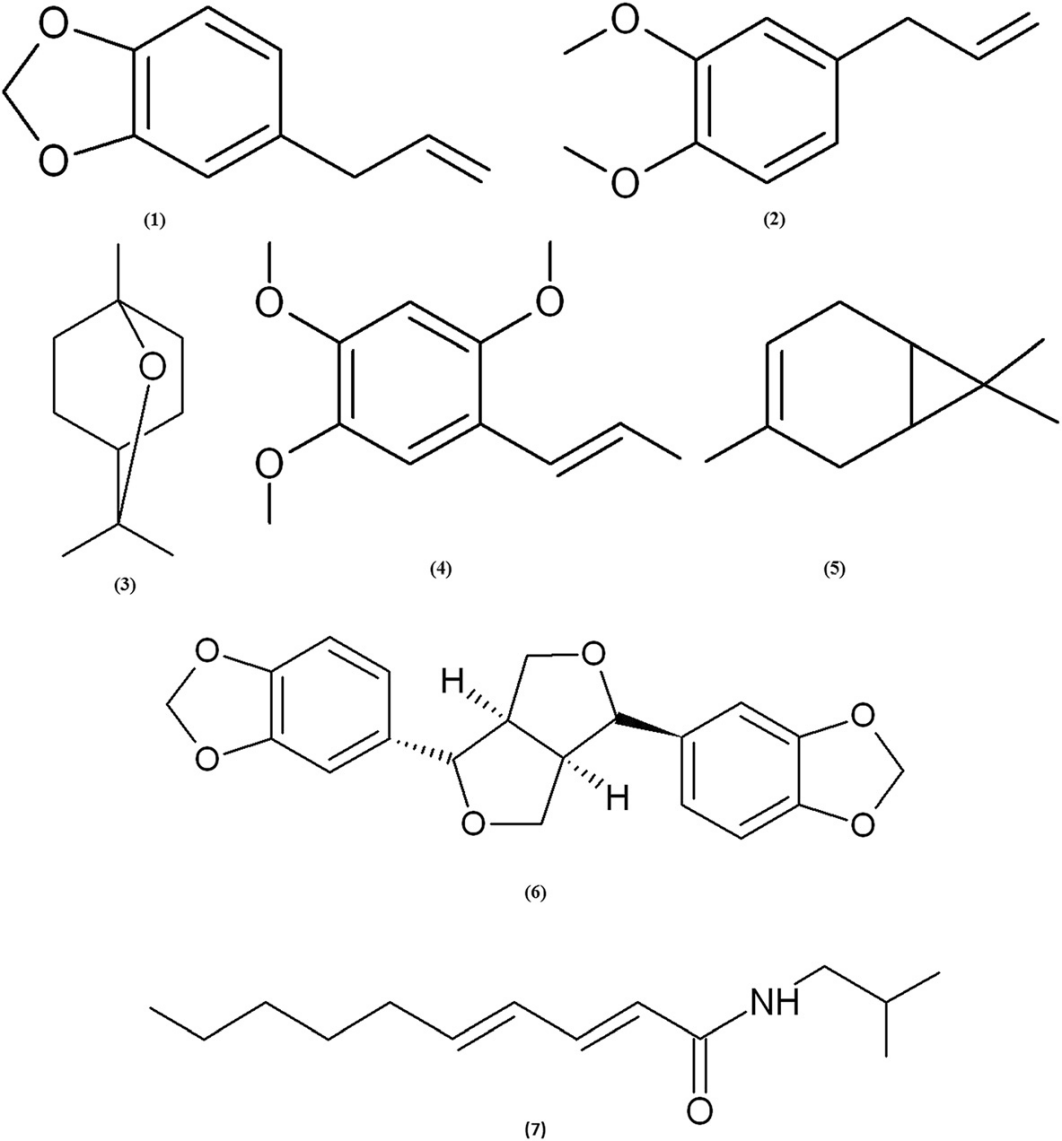
**Active principles of *Asarum heterotropoides *root.** Structures of safrole (1), methyleugenol (2), 1,8-cineole (3), α-asarone (4), δ-3-carene (5), (−)-asarinin (6), and pellitorine (7).

Microtiter plate-based assay-guided fractionation of *A. heterotropoides* root extract afforded seven antibacterial principles identified by spectroscopic analyses, including MS and NMR. The seven antibacterial principles were safrole (**1**), methyleugenol (**2**), 1,8-cineole (**3**), α-asarone (**4**), δ-3-carene (**5**), (−)-asarinin (**6**), and pellitorine (**7**) (Figure 1). Safrole (**1**) was identified on the basis of the following evidence: pale yellow viscous oil. UV (EtOH): λ_max_ nm 210, 290. EI-MS (70 eV), *m*/*z* % relative intensity): 162 [M]^+^ (40), 131 (46), 104 (71), 91 (17), 77 (100), 63 (8), 51 (25), 41 (6). ^1^H NMR (CDCl_3_, 600 MHz): δ 3.31 (2H, d, *J* = 6.66 Hz), 5.07 (1H, m), 5.10 (1H, d, *J* = 1.56 Hz), 5.92 (1H, m), 6.01 (1H, s), 6.61 (1H, d, *J* = 3.54 Hz), 6.70 (1H, s), 6.75 (2H, d, *J* = 7.86 Hz). ^13^C NMR (CDCl_3_, 150 MHz): δ 39.8 t, 100.7 t, 108.1 d, 108.3 d, 115.3 t, 121.2 d, 133.8 s, 137.5 d, 145.8 s, 147.6 s. Methyleugenol (**2**): viscous oil. UV (EtOH): λ_max_ nm 254. EI-MS (70 eV), *m*/*z* (rel. int.): 178 [M]^+^ (100), 163 (26.3), 147 (21.4), 135 (5.6), 115 (4.8), 91 (10.9), 77 (5.8). ^1^H NMR (MeOD, 600 MHz) : δ 3.31 (2H, s), 3.78 (6H, s), 4.83 (2H, s), 5.92 (1H, m), 6.70 (1H, d, *J* =1.56 Hz), 6.76 (1H, s), 6.85 (1H, d, *J* =8.16 Hz). ^13^C NMR (MeOD, 150 MHz): δ 39.8 t, 56.1 q, 56.1 q, 112.3 d, 114.1 d, 115.9 t, 122.3 d, 133.2 s, 136.5 d, 146.8 s, 149.7 s. 1,8-Cineole (**3**): colorless oil. UV (EtOH): λ_max_ nm 210. EI-MS (70 eV), *m*/*z* (% relative intensity): 154 [M]^+^ (23), 139 (19), 126 (5), 111 (25), 108 (29), 93 (56), 71 (36), 69 (28), 43 (100). ^1^H NMR (CDCl_3_, 600 MHz): δ 1.09 (3H, s), 1.29 (6H, s), 1.43 (1H, s) 1.53 (4H, d, *J* = 8.58 Hz), 1.69 (2H, d, *J* = 11.28 Hz), 2.01 (2H, d, *J* = 2.16 Hz). ^13^C NMR (CDCl_3_, 150 MHz): δ 23.8 t, 23.9 t, 25.5 q, 28.8 q, 28.8 q, 37.0 t, 37.1 t, 39.5 d, 71.3 s, 75.6 s. α-Asarone (**4**): viscous oil. UV (EtOH): λ_max_ nm 254. EI-MS (70 eV), *m*/*z* (relative intensity): 208 [M]^+^ (100), 193 (51.8), 177 (10), 165 (7.5), 134 (3.3), 77 (4.9), 69 (1.6). ^1^H NMR (MeOD, 600 MHz): δ 3.32 (3H, d, *J* = 5.4 Hz), 3.73 (3H, s), 3.75 (3H, s), 3.78 (3H, s), 5.03 (1H, s) 5.93 (1H, s), 6.48 (1H, s), 6.72 (1H, s). ^13^C NMR (MeOD, 150 MHz): δ 34.79 q, 56.67 q, 56.96 q, 57.02 q, 99.74 d, 115.3 d, 116.1 s, 121.6 d, 137.8 d, 144.4 s, 149.9 s, 154.5 s. δ-3-Carene (**5**): colorless viscous oil. UV (EtOH): λ_max_ nm 210. EI-MS (70 eV), *m*/*z* (% relative intensity): 136 [M]^+^ (18), 121 (22), 105 (12), 93 (100), 79 (32), 61 (13), 53 (17), 41 (36). ^1^H NMR (MeOD, 600 MHz): δ 0.62 (2H, t, *J* = 8.58 Hz), 0.73 (3H, t, *J* = 8.22Hz), 1.03 (3H, s), 1.78 (3H d, *J* = 1.14 Hz), 2.16 (2H, dd, *J* = 11.0 and 6.84 Hz), 2.34 (2H, d, *J* = 3.72 Hz), 5.24 (1H, s). ^13^C NMR (MeOD, 150 MHz): δ 13.2 d, 16.6 d, 16.7 s, 18.7 q, 20.7 t, 23.6 q, 24.6 q, 28.3 t, 119.4 d, 131.3 s. (−)-Asarinin **(6**): white powder or needle. [α] ^15.6^_D_: –153^o^ (*c* +002; chloroform). UV (EtOH): λ_max_ nm 241. EI-MS (70 eV), *m*/*z* (relative intensity): 354 [M]^+^ (33), 323 (11.3), 203 (14.5), 178 (13.1), 161 (25), 149 (100), 135 (43), 122 (20.4). IR (CDCl_3_) *v*_max_: 2963, 2897, 1500, 1488, 1439, 1371, 1253, 1032, 960, 794, 736. ^1^H NMR (CDCl_3_, 600 MHz): δ 2.84 (2H, dd, *J* = 4.2 and 13.2 Hz), 3.30 (2H, m), 3.84 (2H, m), 4.11 (1H, d, *J* = 11.4 Hz), 4.41 (1H, d, *J* = 6.6 Hz), 5.95 (4H, d, *J* = 7.2 Hz), 6.79 (4H, m), 6.86 (1H, s), 6.88 (1H, s). ^13^C NMR (CDCl_3_, 150 MHz): δ 50.3 d, 54.8 d, 69.8 t, 71.1 t, 82.2 d, 87.8 d, 101.1 t, 101.3 t, 106.6 d, 106.8 d, 108.3 d, 108.4 d, 118.8 d, 119.7 d, 132.4 s, 135.3 s, 146.7 s, 147.4 s, 147.8 s, 148.1 s. Pellitorine (**7**): viscous oil. UV (EtOH): λ_max_ nm 220. EI-MS (70 eV), *m*/*z* (relative intensity): 223 [M]^+^ (45.2), 208 (10), 180 (6.2), 167 (8.5), 152 (100), 113 (9.7), 96 (22.3), 72 (5.8). ^1^H NMR (CDCl_3_, 600 MHz): δ 0.88 (3H, s), 0.91 (3H, s), 0.93 (3H, s), 1.28 (4H, m), 1.37 (2H, d, *J* = 12.0 Hz), 1.75 (2H, m), 2.23 (1H, d, *J* = 6.6 Hz), 3.17 (2H, t, *J* =6.4 and 12.9 Hz), 5.56 (1H, m), 5.76 (1H, m), 6.09 (1H, m), 7.19 (1H, m), 8.03 (1H, s). ^13^C NMR (CDCl_3_, 150 MHz): δ 13.3 q, 20.1 q, 20.1 q, 22.5 t, 28.5 t, 28.6 d, 31.4 t, 32.9 t, 47.1 t, 121.7 d, 128.2 d, 141.3 d; 142.2 d, 166.4 s. The interpretations of proton and carbon signals of compounds **1**, **3**, **5**, and **2**, **4**, **6**, and **7** were largely consistent with those of del Fierro et al. [21], Liu et al. [22], Johnson and Kadow [23], and Perumalsamy et al. [18], and Park et al. [24], respectively.

### Growth inhibitory effect on harmful or nonpathogenic intestinal bacteria

The inhibitory activities of the seven constituents and ciprofloxacin against five harmful and two nonpathogenic intestinal bacteria examined were likewise compared (Table 4). Responses varied according to bacterial species and compound examined. As judged by MIC values, δ-3-carene showed the most potent growth inhibitory activity against four Gram-positive bacteria (0.18–0.70 mg/mL) and two Gram-negative bacteria (0.18–0.70 mg/mL) except for *S. enterica* serovar Typhimurium (2.94 mg/mL). The MIC of methyleugenol was 0.70 and 5.80 mg/mL against *C. difficile* and *C. perfringens*, respectively, and was between 1.47 and 2.94 mg/mL against the other two Gram-positive bacteria and three Gram-negative bacteria. The MIC of 1,8-cineole, pellitorine, and (−)-asarinin was between 1.47 and 2.94 mg/mL against all Gram-positive bacteria (except for pellitorine against *C. perfringens* (0.70 mg/mL)) and three Gram-negative bacteria. The MIC of α-asarone was between 1.47 and 2.94 mg/mL against two Gram-positive bacteria except for *C. paraputiricum* (5.80 mg/mL) and *C. perfringens* (12 mg/mL) and three Gram-negative bacteria. The MIC of safrole was 2.94 mg/mL against three Gram-positive bacteria except for *C. paraputiricum* (23.5 mg/mL) and was 1.47, 7.20, and 12 mg/mL against *B. fragilis*, *E. coli*, and *S. enterica* serovar Typhimurium, respectively. Overall, all of the constituents were less potent at inhibiting microbial growth than ciprofloxacin against all Gram-positive bacteria (MIC, 0.063–0.25 mg/mL) and three Gram-negative bacteria (MIC, 0.063–0.125 mg/mL).

**Table 4:** **Minimal inhibitory concentrations (MICs) of *Asarum heterotropoides *root constituents against five harmful and two nonpathogenic intestinal bacteria using a microtiter plate-based antibacterial bioassay**

	**MIC (mg/mL)**						
	**Gram-positive bacteria**^**a**^				**Gram-negative bacteria**^**b**^		
**Material**	**Cd**	**Cm**	**Cp**	**Sa**	**Bf**^**c**^	**Ec**^**c**^	**St**
δ-3-Carene	0.70 cB	0.70 eB	0.70 dB	0.18 cC	0.70 cB	0.18 dC	2.94 bA
Methyleugenol	0.70 cD	1.47 dC	5.80 bA	2.94 aB	2.94 aB	1.47 cC	1.47 cC
1,8-Cineole	1.47 bB	1.47 dB	2.94 cA	1.47 bB	2.94 aA	2.94 bA	2.94 bA
Pellitorine	2.94 aA	1.47 dB	0.70 dC	1.47 bB	2.94 aA	1.47 cB	1.47 cB
(−)-Asarinin	2.94 aA	2.94 cA	2.94 cA	2.94 aA	2.94 aA	2.94 bA	1.47 cB
α-Asarone	2.94 aC	5.80 bB	12.0 aA	1.47 bD	1.47 bD	2.94 bC	1.47 cD
Safrole	2.94 aD	23.50 aA	2.94 cD	2.94 aD	1.47 bE	7.20 aC	12.0 aB
Ciprofloxacin	0.25 dA	0.125 fB	0.125 eB	0.063 dC	0.125 dB	0.063 eC	0.063 dC

Means in a column followed by the same lowercase letter and means in a row followed by the same uppercase letter are not significantly different (P > 0.05; Bonferroni test).

^a^Cd, *C. difficile* ATCC 9689; Cm, *C. paraputiricum* ATCC 25780; Cp, *C. perfringens* ATCC 13124; Sa, *S. aureus* ATCC 12600.

^b^Bf, *B. fragilis* ATCC 25285; Ec, *E. coli* ATCC 11775; St, *S. enterica* serovar Typhimurium ATCC 13311.

^c^Nonpathogenic bacteria.

### Growth inhibitory effect on beneficial intestinal bacteria

The growth inhibitory effects of all compounds on six lactic acid-producing bacteria (four bifidobacteria and two lactobacilli) and one acidulating bacterium (*C. butyricum*) were likewise compared (Table 5). Responses varied according to bacterial species and compound examined. Based on MIC values, pellitorine showed the most potent growth inhibitory activity against all test bacteria (MIC, 1.47–5.80 mg/mL). However, the constituent was less potent at inhibiting microbial growth than ciprofloxacin against all bacteria (MIC, 0.031–0.063 mg/mL). The MIC of (−)-asarinin was between 1.47 and 2.94 mg/mL against *B. longum*, *L. casei*, and *C. butyricum* and was 5.80 mg/mL against the other four bacteria. Except for *C. butyricum* (MIC, 0.7 mg/mL) and *B. infantis* (5.80 mg/mL), the MIC of δ-3-carene was 12 mg/mL against the other five bacteria. The MIC of α-asarone, 1.8-cineole, and methyleugenol was between 1.47 and 2.94 mg/mL against *B. breve*, *B. longum*, and *L. casei* (except for α-asarone and methyleugenol against *B. breve* (MIC, 5.80 mg/mL)) and was between 12 and 23.50 mg/mL against the other four bacteria. The toxicity of safrole was the lowest of any of the constituents.

**Table 5:** **Minimal inhibitory concentrations (MICs) of *Asarum heterotropoides *root constituents against seven beneficial Gram-positive intestinal bacteria using a microtiter plate-based antibacterial bioassay**

	**MIC (mg/mL)**						
**Compound**	**Bb**^**a**^	**Bi**^**a**^	**Be**^**a**^	**Bl**^**a**^	**La**^**a**^	**Lc**^**a**^	**Cb**^**b**^
δ-3-Carene	12.0 cA	5.80 cB	12.0 aA	12.0 aA	12.0 bA	12.0 aA	0.70 eC
Methyleugenol	23.50 aA	23.50 aA	5.80 bC	1.47 cD	12.0 bB	1.47 cD	23.50 aA
1,8-Cineole	23.50 aA	12.0 bB	2.94 cC	2.94 bC	12.0 bB	2.94 bC	12.0 bB
Pellitorine	2.94 eB	5.80 cA	2.94 cB	1.47 cC	2.94 dB	1.47 cC	1.47 dC
(−)-Asarinin	5.80 dA	5.80 cA	5.80 bA	1.47 cC	5.80 cA	1.47 cC	2.94 cB
α-Asarone	12.0 cA	12.0 bA	5.80 bB	1.47 cC	12.0 bA	1.47 cC	12.0 bA
Safrole	22.70 bB	23.50 aA	12.0 aC	12.0 aC	22.70 aB	12.0 aC	23.50 aA
Ciprofloxacin	0.031 fB	0.031 dB	0.031 dB	0.063 dA	0.063 eA	0.063 dA	0.031 fB

Means in a column followed by the same lowercase letter and means in a row followed by the same uppercase letter are not significantly different (P > 0.05; Bonferroni test).

^a^Lactic acid-producing bacteria: Bb, *B. bifidum* ATCC 29521; Bi, *B. infantis* ATCC 25962; Be, *B. breve* ATCC 15700; Bl, *B. longum* ATCC 15707; La, *L. acidophilius* ATCC 4356; Lc, *L. casei* ATCC 393.

^b^Acidulating bacterium: Cb, *C. butyricum* ATCC 25779.

### Effect on morphostructures of test microorganisms

**Figure 2:**
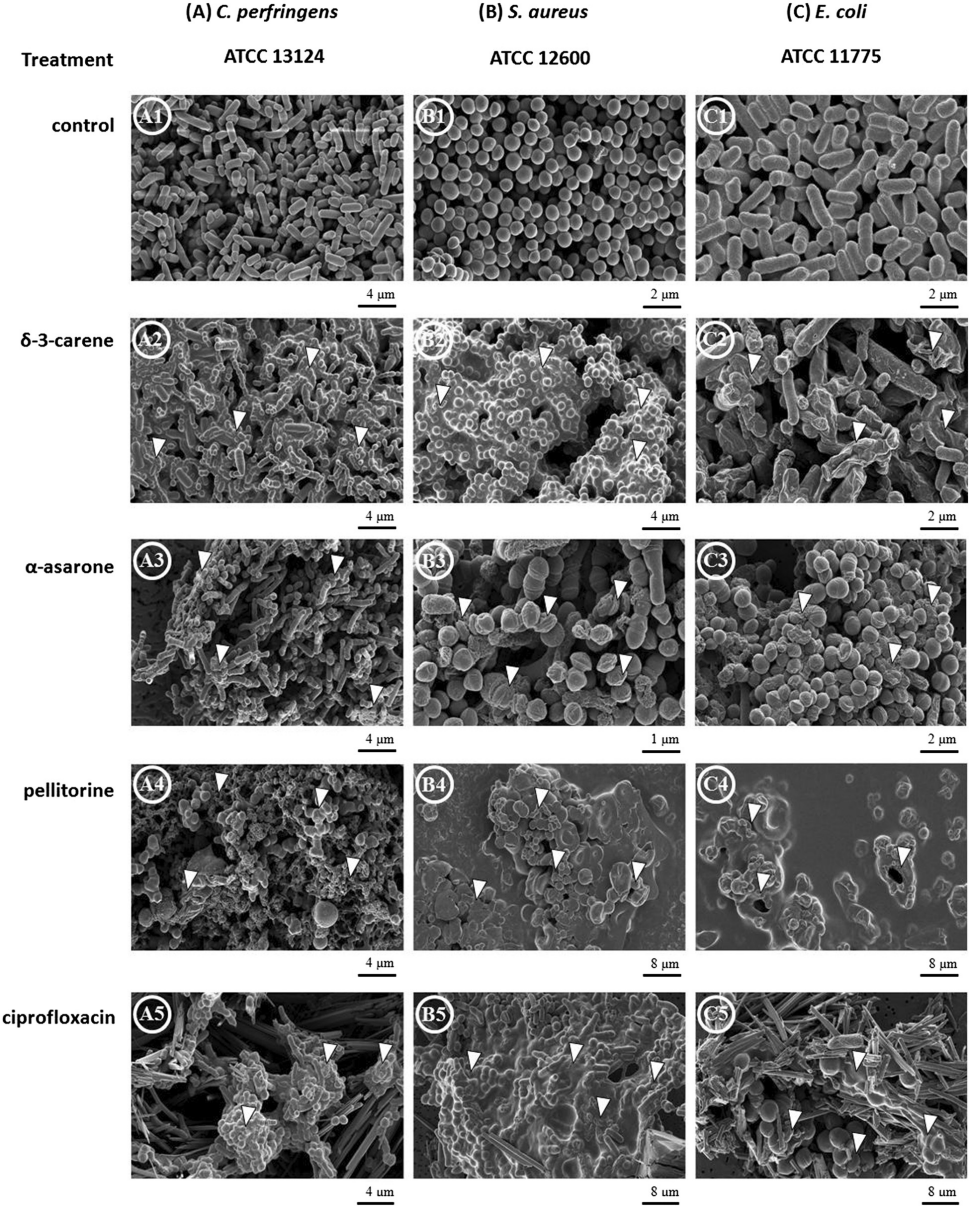
**Effect on morphostructural changes in test microorganisms.** Scanning electron micrographs of two Gram-positives **(A)***Clostridium perfringens* ATCC 13124 and **(B)***Staphylococcus aureus* ATCC 12600 and a Gram-negative **(C)***Escherichia coli* ATCC 11775 without or with treatment of the test compounds. White triangles indicate the morphological changes in cells of the test bacteria treated with corresponding compounds: **A1**, untreated, well divided and rod-shaped cells; **A2**, undivided, growth inhibited cells; **A3**, undivided, growth inhibited cells; **A4**, cell wall and cytoplasmic membrane disruption; **A5**, growth inhibited cells with cytoplasmic membrane disruption. **B1**, untreated, smooth and spherical shaped cells; **B2**, undeveloped cells with cytoplasmic membrane disruption; **B3**, cells with damaged cell membrane surface; **B4**, growth inhibited cells with membrane disruption; **B5**, undivided cells with collision. **C1**, untreated, well divided elongated cells; **C2**, wrinkled shaped cells; **C3**, growth inhibited cells; **C4**, growth inhibited cells with membrane disruption; **C5**; growth inhibited cells with collision.

The morphostructural effects of δ-3-carene, α-asarone, or pellitorine treated with 2 times the MIC of each strain on two selective Gram-positive bacteria (*C. perfringens* ATCC 13124 and *S. aureus* ATCC 12600) and one Gram-negative bacteria (*E. coli* ATCC 11775) were compared with those of ciprofloxacin treated with 2 times the MIC of each strain. Untreated cells of *C. perfringens*, *S. aureus*, and *E. coli* were intact and showed a normal smooth surface (Figure 2 A1, B1, and C1). The scanning electron micrographs for *C. perfringens*, *S. aureus*, and *E. coli* treated with δ-3-carene showed inhibition of cell growth with undivided cells (Figure 2 A2), large surface collapse along with cell membrane damage (Figure 2 B2), and abnormality with wrinkled cells (Figure 2 C2), respectively. In the bacterial strains treated with α-asarone, inhibition of cell growth with undivided cells in *C. perfringens* (Figure 2 A3), disruption and lysis of cell membrane in *S. aureus* (Figure 2 B3), and conversion of the rod to coccoid form in *E. coli* (Figure 2 C3) were observed. Most of cells were clustered and stuck to each other. In the three bacterial strains treated with pellitorine, cell wall and cytoplasmic membrane were completely destroyed and intracellular materials were extruded (Figure 2 A4, B4, and C4). The SEM images for *C. perfringens*, *S. aureus*, and *E. coli* treated with ciprofloxacin showed destroyed cell wall (Figure 2 A5, B5, and C5). Unlike pellitorine, conversion of the rod to the coccoid form was observed in the ciprofloxacin-treated *C. perfringens* and *E. coli*. The coccoid-shaped cells were clustered and stuck to each other.

## Discussion

Among the intestinal microorganisms, clostridia are possible causative agents of a variety of detrimental conditions involving toxicity, mutagenesis, carcinogenesis, and accelerating aging by transforming a variety of ingested or endogenously formed compounds to harmful agents within the intestinal tract [4,6,7]. On the contrary, bifidobacteria are often taken as useful indicators of human health under most environmental conditions, on the basis that they play important roles in metabolism such as amino acid and vitamin production, aid defense against infection, are associated with longevity, antitumor activities, pathogen inhibition, and immunopotentiation [2,3,5]. It would, therefore, be desirable to both inhibit the growth of potential pathogens and/or increase the numbers of bifidobacteria in the human intestine. For example, extracts of ginseng (*Panax ginseng*) root [25] and green tea (*Thea sinensis*) leaves [26] have been shown to not only enhance the growth of bifidobacteria but also to selectively inhibit various clostridia. In vivo investigations using human volunteers have shown that intake of ginseng extract [27] or green tea polyphenols [28] favorably affected fecal microbiota and biochemical aspects of feces.

In the current study, the antibacterial principles of *A. heterotropoides* root were proved to be the phenylpropanoids safrole (**1**), methyleugenol (**2**) and α-asarone (**4**); the monoterpenoids 1,8-cineole (**3**) and δ-3-carene (**5**); the lignan (−)-asarinin (**6**); the isobutylamide alkaloid pellitorine (**7**). These constituents exhibited growth inhibition of both harmful Gram-positive bacteria and Gram-negative bacteria examined. Overall, all of the constituents were less potent at inhibiting microbial growth than ciprofloxacin. However, the four bifidobacteria, two lactobacilli, and one acidulating bacterium were less sensitive and more susceptible than the harmful bacteria to the constituents and to ciprofloxacin, respectively. This original finding indicates that the *A. heterotropoides* root-derived materials may hold promise for the development of novel and effective antibacterial agents. Similar selective growth responses were reported in *Artemisia princeps* var. *orientalis* containing the sesquiterpene lactones seco-tanapartholide A and B [29].

Investigations on the modes of action of naturally occurring antibacterial agents provide practically important information for the control of bacterial diseases, such as the most appropriate formulations and delivery means to be adopted for their future commercialization, and for the development of selective control alternatives with novel target sites and low toxicity [30,31]. The modes of action of plant secondary substances such as alkaloids, phenolics, and terpenoids have been well reviewed by Wink [11]. Major targets (activities) include biomembrane (e.g. membrane disruption or inhibition of membrane protein), proteins (covalent or noncovalent bonding), specific interaction (e.g. inhibition of enzymes or ion pumps protein biosynthesis), and DNA (e.g. inhibition of DNA topoisomerase I or inhibition of transcription) [11]. For example, the monoterpene alcohols citronellol and geraniol appear to cause gross cell wall and cytoplasmic membrane damage and provoke lysis of *Streptococcus pneumoniae*[32], whereas the terpene oxide 1,8-cineole causes the leakage of 260-nm-light-absorbing material and renders cells susceptible to sodium chloride [33]. The anti-*Staphylococcus aureus* activity of the terpene phenol thymol is due to damage in membrane integrity, which further affects pH homeostasis and equilibrium of inorganic ions [34]. In addition, the most bioactive plant secondary substances are known to be more toxic against Gram-positive bacteria than Gram-negative bacteria [35-37]. It has been suggested that the tolerance of Gram-negative bacteria to antibacterial substances is related to the hydrophilic surface of their outer membrane, functioning as a barrier to the penetration of various molecules, and is also associated with the enzymes in the periplasmic space, which are capable of breaking down the molecules [37,38]. However, it suggests that Gram-positive bacteria do not have cell walls [38].

In the current study, SEM observations confirmed different degrees of physical damage and morphological alteration to both Gram-positive bacteria and Gram-negative bacteria treated with δ-3-carene, α-asarone, pellitorine, or ciprofloxacin. δ-3-Carene and α-asarone caused growth inhibition or lysis of cells. Pellitorine and ciprofloxacin caused destruction of the cell wall and cytoplasmic membrane of the three bacterial strains, although the effects on morphostructures were different. These disruptions might cause the loss of shape and integrity of cells which was followed by the cell death. The current finding indicates that the alkaloid, the phenylpropanoid, the terpenoid, and the fluoroquinolone antibiotic do not share a common mode of action as described by Wink [11]. In addition, the beneficial Gram-positive bacteria and the harmful and nonpathogenic Gram-negative bacteria were observed to have different degrees of antimicrobial susceptibility to the constituents, although the antimicrobial susceptibility of the harmful Gram-positive bacteria and the harmful and nonpathogenic Gram-negative bacteria was not observed. Ciprofloxacin susceptibility among the bacterial groups did not differ greatly. Detailed tests are needed to fully understand the different susceptibility of the constituents to bacteria.

## Conclusion

*A. heterotropoides* root-derived preparations containing the constituents described could be useful as potential antibacterial products or lead molecules for the prevention or eradication from humans from diseases caused by harmful intestinal bacteria. The selectivity of the constituents against harmful intestinal bacteria may be an indication of at least one of the pharmacological actions of *A. heterotropoides*. For practical use of the root-derived materials as novel antibacterial products to proceed, further studies are needed to establish their human safety and whether this activity is exerted in vivo after consumption of *A. heterotropoides* root extract and its constituents by humans. In addition, their antibacterial modes of action need to be established and formulations for improving antibacterial potency and stability need to be developed.

## Abbreviations

ATCC: American type culture collection; BHI: Brain heart infusion; CFU: Colony-forming unit; DMSO: Dimethylsulfoxide; EG: Eggerth-Gagnon; HPLC: High-performance liquid chromatograph; MIC: Minimal inhibitory concentration; SEM: Scanning electron microscopy; TLC: Thin-layer chromatography.

## Competing interests

The authors declare that they have no competing interests.

## Authors’ contributions

HP and YJA conceived and designed the experiments. HP, MYJ, and SMH performed the experiments. YJA analyzed the data. HP and YJA wrote the paper. All authors read and approved the manuscript.

## Authors’ information

^1^Research Institute for Agricultural and Life Sciences, College of Agriculture and Life Sciences, Seoul National University, Seoul 151–921, Republic of Korea.

^2^WCU Biomodulation Major, Department of Agricultural Biotechnology, Seoul National University, Seoul 151–921, Republic of Korea.

## Pre-publication history

The pre-publication history for this paper can be accessed here:

http://www.biomedcentral.com/1472-6882/13/245/prepub
